# The short-term recovery of corticomotor responses in elbow flexors

**DOI:** 10.1186/s12868-019-0492-x

**Published:** 2019-03-14

**Authors:** Saied Jalal Aboodarda, Selina Fan, Kyla Coates, Guillaume Y. Millet

**Affiliations:** Human Performance Laboratory, Faculty of Kinesiology, University of Calgary, 2500 University Dr NW, Calgary, AB T2N 1N4 Canada

**Keywords:** Biceps brachii, Brain excitability, Cerebral cortex, Motoneurone, Transcranial magnetic stimulation

## Abstract

**Background:**

The recovery of neurophysiological parameters at various time intervals following fatiguing exercise has been investigated previously. However, the repetition of neuromuscular assessments during the recovery period may have interfered with the true corticomotor excitability responses. In this experiment, fatiguing contractions were combined with a single post-fatigue assessment at varying time points. Ten participants undertook 5 bouts of 60-s maximal voluntary contractions (MVC) of the elbow flexors, separated by 20 min. Before and after each 60-s fatiguing exercise (FAT), participants performed a series of 6-s contractions at 100, 75 and 50% of their MVC during which transcranial magnetic, transmastoid electrical and brachial plexus electrical stimuli were used to elicit motor evoked potentials (MEP), cervicomedullary motor evoked potentials (CMEP) and compound muscle action potentials (Mmax) in the biceps brachii muscle, respectively. Post-FAT measurements were randomly performed 0, 15, 30, 60, or 120 s after each FAT.

**Results:**

MVC force declined to 65.1 ± 13.1% of baseline following FAT and then recovered to 82.7 ± 10.2% after 60 s. The MEP·Mmax^−1^ ratio recorded at MVC increased to 151.1 ± 45.8% and then returned to baseline within 60 s. The supraspinal excitability (MEP·CMEP^−1^) measured at MVC increased to 198.2 ± 47.2% and fully recovered after 30 s. The duration of post-MEP silent period recorded at MVC elongated by 23.4 ± 10.6% during FAT (all *P* < 0.05) but fully recovered after 15 s.

**Conclusions:**

The current study represents the first accurate description of the time course and pattern of recovery for supraspinal and spinal excitability and inhibition following a short maximal fatiguing exercise in upper limb.

## Background

Neuromuscular fatigue (NMF) refers to any progressive exercise-induced change in the central (proximal to the neuromuscular junction) and/or peripheral (at or distal to the neuromuscular junction) systems that reduce the force production capacity of a voluntary or electrically-induced evoked contraction [1]. In order to uncover the influence of exercise on the etiology of NMF development, transcranial magnetic stimulation (TMS) of the cerebral cortex in combination with electrical stimulation of the peripheral motor nerve (PNS) have been used extensively. With these non-invasive techniques, investigators can evaluate the contribution of central and peripheral mechanisms to the development of NMF in the exercised muscles [2]. The specific patterns of NMF development and recovery are dependent upon the nature of the task, the subjects, and the muscle group tested [3–5]. Additionally, the various factors that contribute to fatigue recover at different rates. For instance, the gradual return of maximal voluntary force output (MVC) to baseline (which would take upwards of 10 min) has been attributed to peripheral factors while central factors such as central voluntary activation and corticospinal excitability have been shown to recover much more rapidly [6–8].

In the upper limb, previous studies have assessed the recovery of neurophysiological parameters following fatiguing single limb exercise by repeating neuromuscular evaluations at various time points from 2 s to 20 min post exercise [6, 7, 9–13]. Each neuromuscular evaluation involved multiple maximal or submaximal voluntary contractions; therefore, the repetitive contractions used throughout the recovery period may have altered the recovery pattern of neurophysiological parameters. Indeed, brief upper limb contractions (e.g. 2–6 s contractions at 50% MVC) have been shown to modulate the corticospinal pathway excitability for as long as 15 s [14] to 390 s [15] following the contractions. Some studies have avoided the use of additional contractions during the recovery period by analyzing recovery in the relaxed muscle; however, this method does not elucidate the recovery of central processes contributing to the performance of voluntary contractions [16–18].

The efficacy of the corticomotor pathway in the transfer of central commands (from cerebral cortex to the active muscles) can be evaluated through the electromyographic (EMG) responses to TMS and PNS, known as motor evoked potentials (MEP), and compound muscle action potentials (M wave), respectively [11, 16]. To further differentiate whether alterations in corticospinal excitability are occurring at the supraspinal or spinal motor neuron level, transmastoid electrical stimulation (TMES) of the descending corticospinal tract is used to produce cervico-medullary motor evoked potentials (CMEP) which reflect the excitability of the motoneuron pool [16, 17, 19]. Increases in MEP and CMEP amplitude or area relative to the Mwave, are indicative of heightened excitability of the corticospinal and spinal motoneurons, while the MEP·CMEP^−1^ ratio can be calculated to evaluate supraspinal excitability [16]. An increase in silent period (SP, i.e. a period of EMG silence after MEP) is an indication of corticospinal inhibition [20–23].

MEP amplitude and area as well as the duration of silent period have been shown to increase during fatiguing contractions in upper limb muscles [7–9, 11]. When recovery measurements are taken on a relaxed muscle, the initial MEP facilitation is followed by a long-lasting MEP depression [16, 24, 25]. However, this depression is masked if MEP is measured during brief contractions. During contractions, the increased MEP displays an initial rapid recovery (depression). A complete return to baseline however has been shown to occur as quickly as 15 s following 3-min of intermittent elbow contractions [8], or as slowly as 10 min following repeated 22-s elbow flexor MVC’s [6]. Similar to MEP, CMEP depression during the recovery period is only seen when the muscle is relaxed, whereas a return to baseline occurs within 15 s when CMEP is measured during contractions [16, 18, 26]. The increased SP during fatiguing upper limb exercise also typically recovers within 15–30 s of exercise termination [7, 8, 25, 27], although the exact time course of recovery for this measurement has yet to be established. Again, the repetitive post-fatigue evaluations may have prevented definitive conclusions on the recovery of EMG parameters to be drawn from these studies.

Thus, the current study aimed to specifically examine the recovery time courses of neurophysiological responses to a fatiguing task in the elbow flexors without the potential influence of repetitive post-fatigue assessment. We hypothesize that in the absence of repetitive post-fatigue assessments, the responses elicited by corticospinal stimuli including MEP, CMEP and SP would return to baseline level within a few seconds whereas the recovery of the maximal force output and voluntary activation would take much longer. An accurate estimate of the recovery of these neuromuscular responses (particularly within the first 60 s of recovery) could provide guidelines for the timing of future neuromuscular evaluations.

## Methods

### Subjects

Ten recreationally active male participants (28.7 ± 6.1 year, 178.5 ± 5.2 cm, 75.1 ± 6.8 kg) volunteered to participate in this study. All participants completed the Physical Activity Readiness Questionnaire-Plus form, a checklist of contraindications for TMS [28], and signed the informed consent form before participating in the study. All the participants were right handed based on Edinburgh Handedness Inventory [29]. None of the subjects had a history of musculoskeletal, neurological disease or were taking medications. The study protocol was conducted in accordance with declaration of Helsinki and was approved by the ethics board at the University of Calgary (REB16-0697).

### Experimental protocol

The study was divided into two separate sessions: a familiarization and a testing session. During the familiarization session, the experimental procedure was explained, the written informed consent was obtained and participants were familiarized with the experimental protocol. During the actual testing session, participants were equipped with the EMG and stimulating electrodes. They were then seated in front of the elbow flexion ergometer with the hip, right shoulder and elbow positioned at 90°. The forearm was attached to a force transducer (Model LC101-2K, Omegadyne Inc., Sunbury, OH) with a velcro wrist strap. The force and EMG signals were monitored on a computer screen placed directly in front of the subject.

**Fig. 1:**
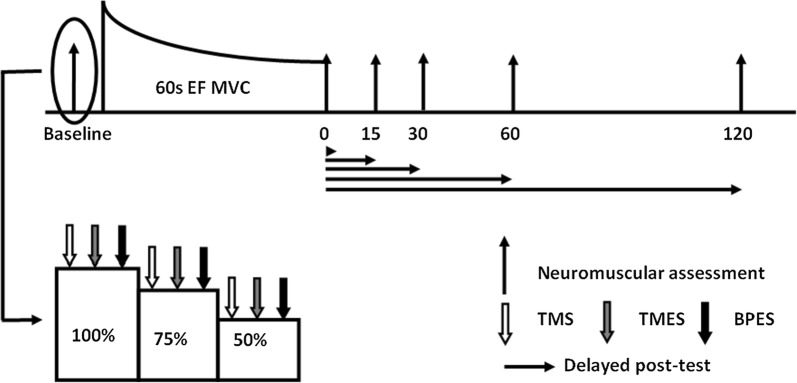
Experimental protocol. Participants undertook five trials of 60-s elbow flexion MVCs (FAT). Before and after each FAT, one neuromuscular function assessment including a series of continuous 6-s elbow flexor contractions at 100%, 75% and 50% of their MVC was performed. During each contraction, transcranial magnetic (TMS), transmastoid electrical (TMES), and brachial plexus electrical stimulation (BPES) were elicited every 2 s. The time points for the post-fatigue measurement  were 0 s, 15 s, 30 s, 60 s, and 120 s, and were selected randomly for each participant

**Fig. 2:**
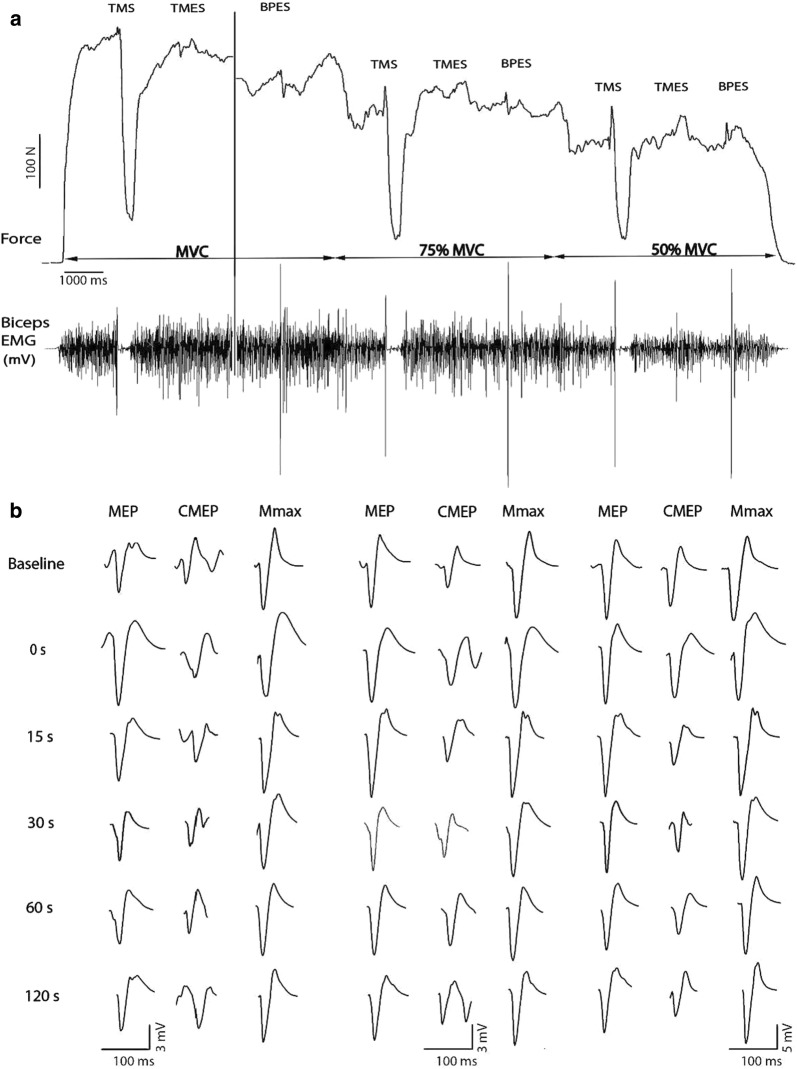
Raw data traces of force output and EMG signals at 100%, 75%, and 50% MVC (**a**). Evoked EMG responses recorded from the biceps brachii muscle of a single subject in response to motor cortical (MEP), spinal (CMEP) and peripheral nerve stimulation (Mmax) at 100%, 75%, and 50% MVC (**b**). The measurements were performed at one of five time points, either 0 s, 15 s, 30 s, 60 s,  or 120 s post-test

Before initiation of the neuromuscular evaluations, the optimal intensity for the PNS was determined (see below). The participants then undertook a warm-up protocol involving three repetitions of elbow flexion at 10%, 30%, 50% and one contraction at 70% of the MVC recorded during the familiarization session. Each warm up contraction was 5 s long with 5 s of rest in between contractions. The warm-up was followed by two 5-s isometric MVCs, with 2 min of rest in between. The determination of TMS and TMES intensities were performed at 20% MVC. Following this, neuromuscular function assessments including a series of TMS, TMES and PNS were elicited every 2-s while the participants performed continuous 6-s elbow flexor contractions at 100%, 75% and 50% of their MVC (Figs. 1, 2). For each assessment, the 75 and 50% of MVC values were immediately calculated after the MVC and displayed on a computer screen using the data acquisition software (LabChart 8), with no rest between contractions at each intensity. The fatigue protocol consisted of a 60-s elbow flexion MVC (FAT). The FAT was repeated 5 times every 20 min. Before and after each FAT, one neuromuscular function assessment was performed. The time-delay for the post-fatigue measurement was 0, 15, 30, 60, or 120 s, and was selected randomly for each participant.

### Electromyography (EMG)

EMG activity of the biceps and triceps brachii muscles was recorded with pairs of self-adhesive surface electrodes (10-mm recording diameter; Meditrace 100, Covidien, Mansfield, MA) in a bipolar configuration with 20-mm inter-electrode distance. A reference electrode was placed on the lateral epicondyle. Prior to placing the electrodes, the area of skin was shaved, abraded with sandpaper, and cleansed with an isopropyl alcohol swab to decrease skin resistance. An inter-electrode impedance of < 5 kΩ was obtained prior to recording to ensure an adequate signal-to-noise ratio. Signals were converted from analog to digital at a sampling rate of 2000 Hz using a PowerLab data acquisition system (16/35, ADInstruments, Bella Vista, Australia) and an octal bio-amplifier (ML138, ADInstruments; common mode rejection ratio = 85 dB, gain = 500) with a bandpass filter (5– 500 Hz) (ADInstruments). Data was then analyzed offline using LabChart 8 software (ADInstruments).

### Transcranial magnetic stimulation (TMS)

The MEP responses of the biceps and triceps brachii muscles were elicited using a Magstim 200 stimulator (Magstim Company, UK) with a 110-mm double-cone coil (maximum output of 1.4 T) to preferentially stimulate the left motor cortex. Subjects wore a lycra swim cap on which intersecting lines were drawn to identify the vertex using the distance from nasion to inion and from left to right tragus. Every centimetre was demarcated along the nasal-inion line from the vertex to 2 cm posterior to the vertex, as well as laterally to 3 cm over the left motor cortex (6 positions). At each of these points, a stimulus was delivered at 50% maximal stimulator output during voluntary contractions at 20% MVC [30]. The TMS hotspot was defined as the site where the largest MEP amplitude was evoked (on top of a contraction at 20% of MVC). The position of the coil was drawn directly onto the swim cap and kept constant throughout the protocol. Participants performed brief contractions at 20% MVC whereby a superimposed TMS was delivered at 30%, 40%, 50%, 60%, 70% and 80% of maximum stimulator output in random order. Four contractions were performed at each stimulus intensity with a 15-s rest interval between contractions. The intensity which showed the highest superimposed twitch (SIT), with largest MEP for biceps brachii and smallest MEP for triceps brachii, was chosen as TMS intensity for the rest of the testing session [30]. The group mean stimulation intensity was 56 ± 9% of maximum stimulator output. The MEP amplitude recorded at this TMS intensity could be differentiated from the background EMG, during 100% MVC contractions. This stimulation intensity was then used for the remainder of the experiment.

### Transmastoid electrical stimulation (TMES)

CMEP responses were evoked by passing a high-voltage electrical current between surface electrodes placed over the skin on the left (cathode) and right (anode) of the mastoid processes (stimulator Model DS7AH; Digitimer, Welwyn Garden City, Hertfordshire, UK). The stimuli intensity (pulse duration: 100 μs; 400 V maximum) was adjusted to produce CMEP amplitude that matched the MEP amplitude during brief 20% of MVC contraction. In other words, the amplitude of CMEP was matched with the MEP amplitude obtained from the TMS stimulus-responses curve explained above. The group mean stimulation intensity was 218 ± 92 mA.

### Peripheral nerve stimulation (PNS)

Brachial plexus electrical stimulation was used to determine the size of the maximal compound muscle action potential (Mmax) of the biceps and triceps brachii. The stimulating electrodes (Ag–AgCl discs, 20-mm diameter) were placed on the supraclavicular fossa (cathode) and on the acromion process (anode). High-voltage percutaneous electrical stimuli (stimulator Model DS7AH; Digitimer) were delivered to the motor axons at the Erb’s point. The stimulation intensity (200-μs pulse duration; 400 V maximum) was increased in incremental steps (20 mA) until a plateau in Mmax and maximal twitch force was achieved at rest. The intensity was then increased by an additional 30% to ensure supramaximal stimulation. This stimuli intensity was used for the remainder of the experimental session. The group mean stimulation intensity was 232 ± 108 mA.

## Data analysis

### FAT contractions

The force output of the elbow flexors and the background root mean square EMG (rmsEMG) of the biceps brachii muscle were assessed for the first, middle and last 5-s intervals of FAT in the 5 experimental conditions.

### Neuromuscular evaluations

The force and the background EMG [rmsEMG of the biceps and triceps brachii muscles] were quantified over 500 ms prior to each MEP to ensure that changes in corticospinal responses measured at similar target forces were not due to differences in muscle activity prior to the stimulus. The maximum rmsEMG values were normalized to the amplitude of Mmax recorded during the same contraction to produce rmsEMG·Mmax^−1^ ratio (rmsEMG_100_, rmsEMG_75_, rmsEMG_50_).

The peak-to-peak amplitude and area under the signals were measured for each MEP, CMEP and Mmax signal recorded from biceps and triceps brachii muscles. Since the amplitude and area of the signals showed the same results, the area has been reported. In addition, the CMEPs recorded from triceps were not distinguishable from background EMG for majority of the participants, therefore these values were not reported. Onset of MEP, CMEP and Mmax were defined as the point at which the voltage trace became tangential to baseline in either the positive or negative direction. Because the Mmax can change as a result of the level of voluntary activation and fatigue, responses to biceps and triceps MEP and biceps CMEP were normalized to the subsequent Mmax recorded during the same contraction to produce MEP·Mmax^−1^ ratios (MEP_100_, MEP_75_, MEP_50_) and CMEP·Mmax^−1^ ratios (CMEP_100_, CMEP_75_, CMEP_50_), respectively. The MEP·CMEP^−1^ ratios were calculated for biceps at different contraction intensities (MEP·CMEP_100_^−1^, MEP·CMEP_75_^−1^ and MEP·CMEP_50_^−1^) to identify the changes at the cortical level. The duration (ms) of silent period was assessed for biceps MEPs as the interval from the stimulus artefact to the return of the continuous EMG by visual inspection during 100, 75 and 50% of MVC contractions (SP_100_, SP_75_, SP_50_). The triceps silent period was not reported because of inconsistency of the data.

One of the aims of the present study was to measure voluntary activation using TMS. The amplitude of SITs evoked by TMS during contractions at 100, 75, and 50% MVC were calculated and the *y*-intercept of the linear regression between the SITs was used to quantify the estimated resting twitch [31]. Although the TMS-evoked SITs demonstrated a linear regression at rest (r^2^ > 0.9), the post-FAT regression lines were not linear for some participants. This issue could be attributed to poor motor control during fatigue state. Specifically, several participants were unable to maintain the post-FAT MVC force at a plateau level. In these cases, the TMS was elicited at submaximal force (~ at 95% of peak MVC) which would result in overestimation of SIT amplitudes recorded at MVC. Accordingly, these VA values were removed from the manuscript.

### Statistical analysis

Statistical analyses were computed using SPSS software (version 23.0; SPSS, Inc., Chicago, IL). Assumption of normality (Shapiro–Wilk test) and sphericity (Mauchley test) were tested for all of the dependent variables. If the assumption of sphericity was violated, the corrected value for non-sphericity with Greenhouse–Geisser correction was reported. Firstly, in order to compare the rate of decline in the MVC force and maximum rmsEMG across the five FAT trials, a two-way repeated measure analysis of variances (ANOVA) was performed, i.e. time (first, middle and last 5-s of each 60-s contraction) × five experimental conditions. Secondly, in order to ensure that there was no significant difference between the five randomized experimental conditions at baseline, one-way repeated measures ANOVA was run on the raw data for all variables. To measure the potential cumulative effect of the 5 × 60-s sustained MVCs performed in the experimental session, an additional one-way ANOVA was conducted on the five baseline measures (independent of the randomized post-fatigue trials). This cumulative effect was measured for all outcome variables. In addition, coefficient of variation (CV) was calculated for the MVC force, corresponding rmsEMG, MEP, CMEP and SP values using the five baseline measures (Table 1). Thirdly, in order to determine the effect of FAT on each variable, a paired *t*-test was used to compare the absolute baseline and 0 s values. Finally, the values obtained from measurements at each recovery time point were normalized to the corresponding baseline measure performed before each FAT trial. A one-way repeated measures ANOVA was conducted on five normalized data points to determine the influence of elapsed time on the recovery of MVC force, TMS-induced SIT, rmsEMG, corticospinal excitability and SP measures at 100, 75 and 50% of MVCs contractions. If a significant effect was obtained from the ANOVAs, Bonferroni corrected paired sample t-tests were performed to compare different time points. Significance was defined as P = 0.05.

**Table 1:** Coefficient of variation (CV) calculated for all variables across the 5 baseline measurements

	100% MVC	75% MVC	50% MVC
Force (%)	13.3 (5–23)	14.7 (4–24)	12.2 (4–19)
rmsEMG·Mmax^−1^ (%)	17.8 (11–28)	21.8 (9–33)	19.4 (8–27)
MEP·Mmax^−1^ (%)	10.8 (5–19)	7.1 (2–16)	5.8 (3–9)
CMEP·Mmax^−1^ (%)	33.4 (16–56)	25.9 (9–46)	28.7 (13–49)
SP (%)	4.3 (2–7)	5.1 (2–10)	5.9 (3–11)

The CV (range: minimum–maximum) was calculated for the voluntary force output (force), the corresponding root mean square EMG (rmsEMG·Mmax^−1^), the area of motor evoked potential (MEP·Mmax^−1^), cervicomedullary motor evoked potential (CMEP·Mmax^−1^) and the silent period (SP) at different contraction intensities (i.e. 100, 75 and 50% MVC)

## Results

### Baseline measurements

**Fig. 3:**
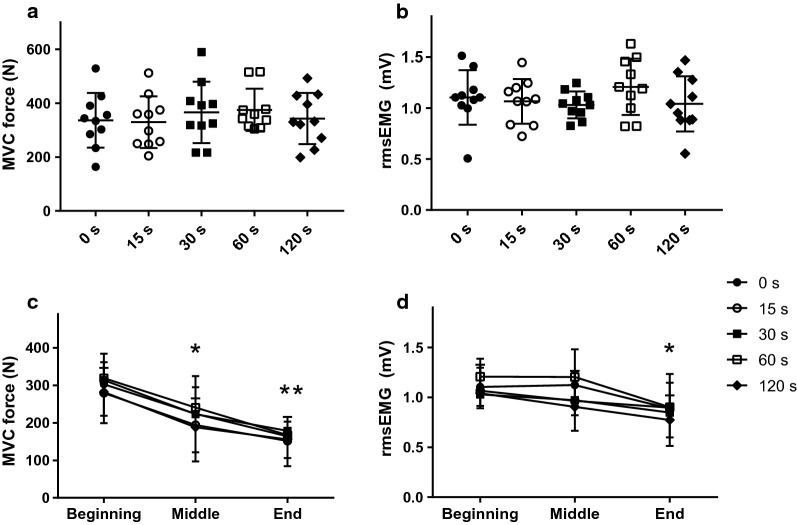
The magnitude of MVC force (**a**) and the corresponding rmsEMG (normalized to Mmax) (**b**) measured during the five pre-FAT trails as well as during the sustained 60-s MVCs (FAT) (**c**, **d**)

No significant differences were observed between the five baseline measures for all variables when the randomization was applied (*P* > 0.2). The individual data for the MVC force and rmsEMG∙Mmax^−1^ are depicted in Fig. 3a. Similarly, no cumulative effect was evident for all outcome measures (*P* > 0.1) except for MVC force which demonstrated a trend towards lower force output at baseline #5 compared to #1 (*P* = 0.058). The CVs for the force output, rmsEMG·Mmax^−1^, MEP·Mmax^−1^, CMEP·Mmax^−1^ and SP measured at baseline are presented in Table 1. The CVs were also calculated for the MVC force and biceps rmsEMG recorded at the beginning of five FAT trails and were found to be 14.9% and 16.6%, respectively.

### Force and rmsEMG during FAT trials

Force and the corresponding rmsEMG measured during the sustained 60-s MVC declined to 54.8 ± 12.2% (group mean: 335.7 ± 88.7 to 172.5 ± 52.8 N; time effect: F_2,18_ = 78.21, *P* < 0.001) and 83.9 ± 26.7% (1.06 ± 0.27 to 0.84 ± 0.29 mV; time effect: F_2,18_ = 5.18, *P* = 0.018), respectively (Fig. 3c, d). There was no significant condition or interaction effects between the five trials.

### Force and rmsEMG

**Fig. 4:**
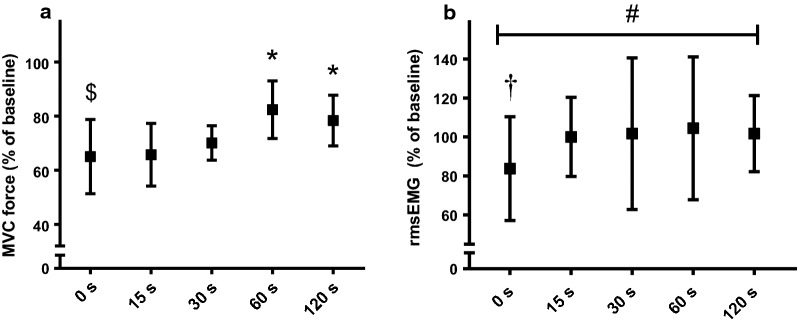
The magnitude of MVC force output (**a**) and rmsEMG_100_ (normalized to Mmax) recorded 500 ms before MEP (**b**) at different time delays following FAT. ^$^Significant difference from Baseline, *significant difference from 0 s, ^†^trend toward significance compared to baseline, ^#^trend toward significance during recovery

The MVC force significantly declined from pre- to post-FAT measurements (to 65.1 ± 13.1% of baseline, *P* < 0.001). MVC showed a significant recovery at 60 s (to 82.4 ± 10.1%, *P* = 0.005) compared to the 0 s time point (Fig. 4a) despite remaining below baseline even at the 120 s measurement (F_4,36_ = 7.95, *P* < 0.001). The rmsEMG_100_ recorded from biceps (500 ms before MEPs) was trending towards a significant decrease from baseline to 0 s, and then to recovery at 15 s (*P* = 0.052, Fig. 4b). The rmsEMG_50_ significantly declined from baseline to 0 s (*P* = 0.001) however ANOVA did not show any significant recovery during the post-FAT time points. No change was observed for rmsEMG_75_.

### Corticospinal excitability

**Fig. 5:**
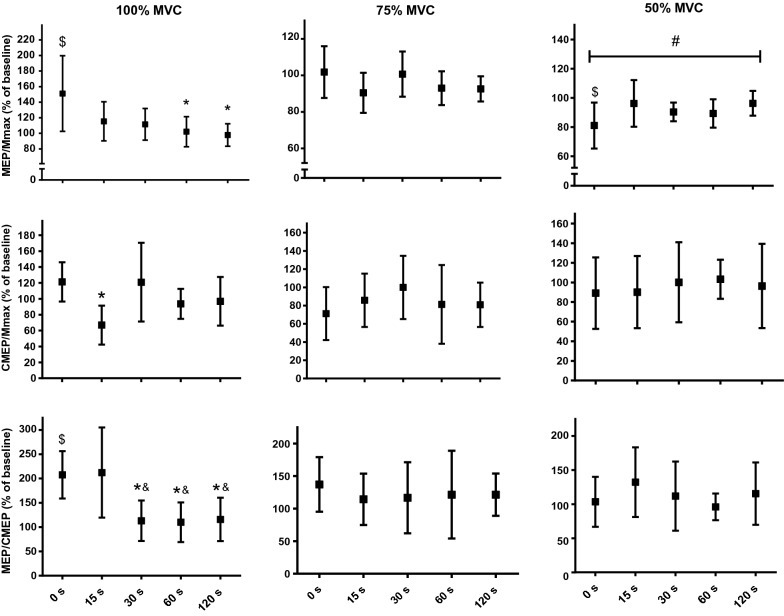
The corticospinal excitability measures (MEP·Mmax^−1^, CMEP·Mmax^−1^ and MEP·CMEP^−1^ ratios) recorded at different contraction intensities and time delays following FAT. ^$^Significant difference from Baseline. *Significant difference from 0 s, ^&^significant difference from 15, ^#^trend toward significance

Biceps MEP_100_ demonstrated a significant facilitation from baseline to 0 s (increased to 151.1 ± 45.8% of baseline, *P* = 0.011) and then gradually returned towards baseline (F_1.7,14.3_ = 6.24, *P* = 0.014) until a significant recovery was displayed at 60 s (*P* = 0.038) (Fig. 5). The MEP_75_ did not show any statistical significance from baseline to 0 s, nor across the recovery time points. The MEP_50_ significantly declined from baseline to 0 s (to 84.6 ± 17.3%, *P* = 0.012). Although the recovery pattern for this parameter showed a trend towards significance (F_4,36_ = 2.56, *P* = 0.060), the Bonferroni corrected paired t-tests did not show any difference between post-FAT time points (Fig. 5). Since MEP values were normalized to Mmax, it is important to report that Mmax area recorded at 100, 75 and 50% of MVC did not show any significance difference between the time delays. The triceps MEP_100_ showed a trend toward a significant decrease (F_1.6,10.1_ = 4.26, *P* = 0.052), however Bonferroni corrected paired t-tests did not show any difference between the recovery time points (Table 2).

CMEP_100_ did not show any difference from baseline to 0 s (*P* = 0.091), however this measure declined significantly from 0 s to 15 s (to 69.3 ± 22.1%, *P* = 0.022). The CMEP_75_ and CMEP_50_ did now show any significant change from baseline to 0 s, nor across the recovery period (Fig. 5).

**Table 2:** Group data (mean and SD) for variables recorded from triceps brachii during the recovery time points

	Baseline	0 s	15 s	30 s	60 s	120 s
Triceps Brachii MEP·Mmax^−1^						
MEP_100_	75.3 (58.0)	46.1 (35.0)	29.8 (14.2)	35.3 (16.8)	38.4 (12.2)	67.8 (37.7)
MEP_75_	50.3 (22.4)	42.8 (33.2)	42.8 (30.5)	29.3 (11.7)	38.1 (31.3)	44.9 (29.6)
MEP_50_	46.7 (18.6)	33.2 (16.6)	39.4 (33.9)	41.3 (29.6)	33.8 (29.0)	36.6 (15.8)
Triceps brachii rmsEMG·Mmax^−1^						
rmsEMG_100_	1.66 (1.02)	1.77 (1.12)	1.32 (0.63)	1.17 (0.60)	1.28 (0.56)	1.94 (0.93)
rmsEMG_75_	1.60 (0.59)	1.40 (0.89)	1.03 (0.60)	1.05 (0.54)	1.03 (0.47)	1.39 (0.88)
rmsEMG_50_	1.09 (0.22)	0.83 (0.35)	0.78 (0.35)	0.71 (0.36)	0.68 (0.26)	1.02 (0.61)

The corticospinal excitability of the triceps muscle (MEP·Mmax^−1^) measured during MVC showed a trend towards significance (*P* = 0.052) whereas it did not show any significance when recorded at 75 and 50% of MVC. The root mean square EMG (rmsEMG·Mmax^−1^) of the triceps muscles recorded before MEP at MVC, 75 and 50% did not show any difference between time points

The MEP·CMEP_100_^−1^ indicated a significant increase in supraspinal excitability from baseline to 0 s (to 198.2 ± 47.2%, *P* < 0.001). During the recovery period, ANOVA showed a significant main effect for this parameter (F_4,24_ = 6.508, *P* < 0.001) where it demonstrated the highest values at 15 s, and then showed a significant return at 30 s (*P* = 0.045) (Fig. 5) The MEP·CMEP_75_^−1^ and MEP·CMEP_50_^−1^ ratios did not show any difference between the six time points.

### Corticospinal inhibition

**Fig. 6:**
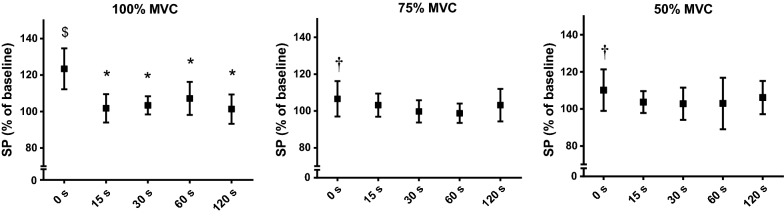
The duration of SP recorded at different contraction intensities and time delays following FAT. ^$^Significant difference from Baseline. *Significant difference from 0 s, ^†^trend toward significance compared to baseline

While FAT resulted in a significant elongation of SP_100_ (to 123.4 ± 10.6%, *P* < 0.001) at 0 s, there was only a trend toward significance for SP_75_ (to 106.6 ± 9.1%, *P* < 0.055*)* and SP_50_ (+110.1% ± 10.6, *P* < 0.052). The SP_100_ returned to baseline at 15 s (*P* < 0.001) (Fig. 6).

## Discussion

The present study is the first to investigate the recovery time course of corticomotor responses in the elbow flexors after a fatiguing contraction without the interference of repeatitive recovery assessments. The main results supported our research hypotheses that (i) the impaired MVC force output partially recovered at 60 s, (2) the facilitated corticospinal and supraspinal excitability (indicated by increased MEP_100_ and MEP·CMEP_100_^−1^, respectively) were recovered at 60 s and 30 s, respectively, and (3) the corticospinal inhibition, indicated by prolonged SP_100_, returned to baseline at 15 s. These findings confirm that measures of NMF and corticospinal excitability and inhibition are transient and recover at different paces. Thus, in order to achieve a precise estimation of the etiology of NMF, neurophysiological measurements should be performed immediately after cessation of the fatiguing task. Another important result of the present study is that the recovery pattern of neurophysiological responses is dependent upon the intensity of the voluntary contraction performed during the assessment.

### MVC force and rmsEMG

Repeated neuromuscular assessments during the recovery period could modulate corticomotor excitability responses; therefore, the present experiment involved FAT trials combined with a single post-fatigue assessment at different time delays. The baseline MVC force output and the corresponding rmsEMG (Fig. 3a, b), as well as the force and rmsEMG at the beginning and termination of FAT contractions (i.e. the first and last 5 s of FATs, Fig. 3c, d) demonstrated similar values between the five randomized trails. These results confirm that the subjects were adequately recovered before the randomized FAT contractions.

Force declined to ~ 65% of baseline at 0 s which is similar to previous studies involving 60-s elbow flexor MVCs [25, 31]. At 60-s post-FAT, maximal force displayed a significant amount of recovery from its minimum value observed at 0 s, but never fully recovered to baseline over the course of measurements. Previous upper limb protocols involving sustained or intermitted isometric contractions (from 90 s to 3 min in duration) have demonstrated that the recovery of maximal voluntary force may take greater than 10 min to return to baseline [6–8]. The present study was not designed to directly discern the recovery pattern of central and/or peripheral mechanisms of fatigue. Yet the changes in rmsEMG100 (Fig. 4b) suggest that alteration in the descending central motor drive might have played a significant role in the reduction and recovery of force output at post-FAT level. On the other hand, those studies which investigated peripheral fatigue mechanisms in upper limb muscles have demonstrated that muscles contractile properties could take greater than 5 min to completely recover following fatiguing isometric contractions [6, 7, 10], very likely explaining why MVC was still 20% below initial values 120 s after exercise cessation. Further research with the single post-fatigue assessment paradigm is required to investigate the time course of full recovery of MVC force as well as central and peripheral mechanisms in upper limb muscles.

### Corticospinal excitability

The MEP_100_ and MEP·CMEP_100_^−1^ ratio was increased at 0 s, whereas no change was observed for the CMEP_100_ at this time point (Fig. 5). These results confirm that the excitability of motor cortical cells is significantly increased during maximal contraction at the termination of FAT [9, 11, 20]. During the next 15 s, the CMEP_100_ declined significantly, however the MEP_100_ and MEP·CMEP_100_^−1^ ratio remained above the baseline (Fig. 5). Gandevia et al., [16] also found an increase in MEP·Mmax^−1^ and MEP·CMEP^−1^ ratio immediately [2–25 s] after a 120-s MVC of the elbow flexors. Contrary to the present study however, these authors measured the corticospinal excitability on relaxed muscles and found a decrease in the CMEP·Mmax^−1^ ratio immediately after cessation of the task. Similar to our study, Taylor et al. [11, 32] and McNeil et al. [9] recorded MEP during maximal contraction and found MEP facilitation during a 120-s elbow flexor MVC. They attributed the enhanced MEP after sustained MVC to (1) a greater net excitability of the corticomotor cells as well as a decrease in refractoriness of these neurons which may increase descending excitatory volleys and/or (2) a decreased disynaptic inhibition at the spinal level. These mechanisms have been suggested to be compensatory pathways adopted by corticomotor circuitries to minimize deterioration of maximal muscle performance  by increasing net excitatory motor drive to the spinal motoneurone pool. Nonetheless, contrary to the present work in which the recovery assessment began immediately following fatiguing exercise, the previously mentioned investigations initiated their post-fatigue cortical stimulations 15 s [9], 30 s [11] and 45 s [32] after the end of the sustained contraction, whereby MEP did not show any significant difference compared to baseline. Considering that an increase in MEP area during the sustained maximal elbow flexion is a well-established concept [8, 9, 11, 32], the failure of prior investigations in observing a residual MEP facilitation at 15, 30 and 45 s supports our argument that starting the post-fatigue assessments with a minimum delay provides a more accurate estimation of the corticospinal modulations.

Contrary to MEP_100_ facilitation, the corticospinal excitability recorded during submaximal contractions (i.e. MEP_50_) demonstrated a significant depression at 0 s. Very few studies have recorded the post-fatigue corticospinal responses during various maximal and submaximal contractions (e.g. contractions at 100, 75 and 50% of MVC); however, in line with this finding, Goodall et al., [33] recently demonstrated a fatigue-induced depression in corticospinal excitability measured during submaximal contraction (10% MVC) in lower limb muscles. The reason for the disparity in MEP responses between maximal and submaximal contraction following FAT is unclear, however given that CMEP_100_ and CMEP_50_ did not demonstrate a significant alteration at 0 s (compared to baseline), it could be postulated that mediation at the motor cortical level was the primary determinant of the excitability of the entire corticospinal pathway (as indicated by the size of MEP). Indeed, supraspinal excitability assessed during MVC (MEP·CMEP_100_^−1^ ratio) showed significant facilitation at 0 and 15 s while no change was observed at 50% of MVC. The rmsEMG_50_ significantly declined from baseline to 0 s, thus it is plausible that a decrease in the magnitude of excitatory cortical volleys could have contributed in MEP_50_ depression. However, a trend towards a lower cortical discharge rate during MVC (indicated  by rmsEMG_100_) could have artificially increased MEP_100_,as it reduces the likelihood of stimulating the corticomotoneuronal cells during the refractory period. Nonetheless, our results indicate that the mechanisms contributing to the increase in MEP_100_ and/or decrease in MEP_50_ after FAT are transient and recover quickly.

The CMEP_100_ initially demonstrated a non-significant facilitation at 0 s and then a significant depression at 15 s. As mentioned earlier, previous investigations that measured CMEP at rest demonstrated a long-lasting depression for this measure following maximal contractions (from 5 to 120 s). As such, it has been suggested that the intrinsic property of the spinal motoneurones becomes less responsive with fatigue [16, 18, 26, 34]. However, it has also been shown that the depression is typically masked when CMEP is recorded during voluntary contraction [11]. Accordingly, we  suggest that the deteriorated  responsiveness of the spinal motoneurones at 0 s could have been masked by descending excitatory cortical input to the motoneurone pool. Considering that this descending excitatory cortical volley  would also be present during the MVC at 15 s, it is unclear why CMEP_100_ decreased at this time point. It is unlikely that this phenomenon was due to a decline in the magnitude of excitatory input to the motoneurone pool. Indeed, it has been shown that withdrawal of excitatory input (disfacilitation) during maximal contraction would increase the responsiveness of the motoneurones and the amplitude of CMEP [35, 36]. Thus, the decrease in CMEP_100_ at 15 s could be associated with inhibitory signalling pathways diverging to the motoneurones, such as through the activation of spinal inhibitory interneurons [37]. As mentioned earlier, Taylor et al. [32] suggest that the reduction of spinal inhibition could be a contributing factor in facilitation of MEP at the end of sustained contractions. We speculate that the restoration of inhibition from 0 to 15 s could not only partially recover (decrease) the facilitated MEP_100_ (Fig. 5), but also depress CMEP_100_ at this time point.

### Corticospinal inhibition

The SP_100_ was prolonged following FAT, however it fully recovered within 15 s of rest. The SP lengthens with fatigue leading to the conclusion that an augmented level of inhibition is built up in the corticomotor circuitries [8, 20]. The increase in duration of SP has been demonstrated following both sustained [9, 11] and intermittent isometric elbow flexor MVCs [8]. The time course of complete recovery for this parameter has been reported as early as 30 s [11], 15 s [9], and 10 s [8] following the cessation of the isometric and intermittent contractions. Therefore, our data supports previous work indicating that SP recovers rapidly, and is not directly associated with motor cortical excitability, which increases after fatigue [8, 11]. SP is mediated through increasing activity of inhibitory interneurons releasing the neurotransmitter gamma-aminobutyric acid (GABA), and subsequent GABA_B_ receptors activation [38, 39]. However, its mechanisms appear to be distinct from short interval intracortical inhibition which is mediated by GABA_A_ inhibitory systems [33, 40, 41]. The data in the present study demonstrated only a trend toward significance for elongation of SP during submaximal contractions (i.e. SP_75_ and SP_50_). Although these results suggest that near maximal contractions are required to observe a prolongation of the SP [11, 20], Goodall et al., [33] found a significant elongation of SP at 10% of MVC. Therefore, further research is necessary to evaluate mechanisms of SP recovery at different contraction intensities following fatigue.

There are several limitations to this study. As with all fatigue studies, the findings should be interpreted by taking the task specificity of fatigue mechanisms into consideration. To avoid contraction-induced fatigue, or modulations in corticomotor responses, only one neuromuscular fatigue assessment was performed before and after each fatiguing contraction. Previous investigations demonstrated that voluntary motor cortical outputs recovered within intervals varying from 15 s to 4 min [6–8], therefore we chose to record the neurophysiological parameters up to 120 s post-test. However, the recovery intervals chosen to represent this timeframe (i.e. 0, 15, 30, 60 and 120 s) were not ideal for the tracking of the changes in corticospinal excitability. Ideally neuromuscular assessments should have been performed after very short rest periods (e.g. 2, 5 and 10 s) to better analyze the patterns of corticospinal recovery. However, adding more fatiguing contractions was not feasible for this project. Finally, further experiments are required that include a control condition in which neurophysiological responses are quantified following repetitive neuromuscular evaluation (i.e. without fatiguing contractions).

## Conclusion

The present study is the first to describe the time course of neuromuscular recovery following a short and maximal fatiguing exercise without the interference of repetitive post-fatigue assessments. We suggest that post-fatigue assessments should be initiated immediately following task cessation because corticospinal excitation and inhibition recover substantially within 30 s of recovery. Although the reasons are not clear, the present study also shows that the intensity of contraction influences corticospinal excitability and inhibition. Whether or not these findings apply to whole-body, dynamic exercises must be confirmed by using an innovative ergometer recently developed in our laboratory [42].

## Abbreviations

CMEP: cervico-medullary motor evoked potentials
CMEP: CMEP·Mmax^−1^ ratio
EMG: electromyographic
FAT: 60-s elbow flexion MVC
MEP: motor evoked potentials
MEP: MEP·Mmax^−1^ ratio
MVC: maximal voluntary force output
NMF: neuromuscular fatigue
PNS: peripheral motor nerve
rmsEMG: root mean square EMG·Mmax^−1^ ratio
SP: silent period
TMES: transmastoid electrical stimulation
TMS: transcranial magnetic stimulation
